# Treatment persistence of biologics among patients with psoriatic arthritis

**DOI:** 10.1186/s13075-021-02417-x

**Published:** 2021-01-29

**Authors:** Amir Haddad, Tal Gazitt, Ilan Feldhamer, Joy Feld, Arnon Dov Cohen, Idit Lavi, Faten Tatour, Irena Bergman, Devy Zisman

**Affiliations:** 1 Rheumatology Unit, Carmel Medical Center, 7 Michal Street, Haifa, Israel; 2 Chief Physician’s Office, Central Headquarters, Clalit Health Services, Tel Aviv, Israel; 3 Siaal Research Center for Family Medicine and Primary Care, Faculty of Health Sciences, Ben-Gurion University of the Negev, Beer-Sheba, Israel; 4 Biostatistics unit, Carmel Medical Center, Haifa, Israel; 5 Internal Medicine Department, Carmel Medical Center, Haifa, Israel; 6 Ruth and Bruce Rappaport Faculty of Medicine, Technion-Israel Institute of Technology, Haifa, Israel

**Keywords:** Psoriatic arthritis, Drug persistence, Biologics

## Abstract

**Background:**

Persistence of biologic therapy in psoriatic arthritis (PsA) patients is an important factor in individualized patient treatment planning and healthcare policy and guideline development.

**Objective:**

To estimate the persistence of biologic agents prescribed to PsA patients in a real-life setting as well as factors associated with improved biologic drug survival in these patients.

**Methods:**

Patients with PsA from a large healthcare provider database with at least two consecutive dispensed prescriptions of a biologic agent indicated for PsA from January 1, 2002, until December 31, 2018, were identified and followed until medication stop date or the end of observation period. Patients were considered non-persistent whenever a permissible lag time of 6 months from the time of prescription issuance until medication filling date was exceeded. Treatment changes were based on physician decisions and patient preferences.

Demographic data including age, sex, body mass index (BMI), ethnicity, smoking history, and socioeconomic status as well as Charlson comorbidity index were retrieved. Data regarding use of steroids and conventional disease-modifying anti-rheumatic drugs (cDMARDs) were also extracted. Descriptive statistics, including means (standard deviations) for continuous variables and frequencies (%) for categorical variables, were used. Persistence estimates were derived using non-parametric survival analysis using Kaplan-Meier functions, with treatment discontinuations as failure events. Cox regression hazard ratio models were conducted to investigate factors associated with drug persistence**.**

**Results:**

A total of 2301 PsA patients with 2958 treatment periods were identified and included in the analyses. Mean age of the study population was 50.9 ± 14 years, 54% were females, 70.4% were with BMI > 25, 40% were current smokers, and 76% were with a Charlson comorbidity index > 1. The most commonly prescribed drug was etanercept (33%), followed by adalimumab (29%), golimumab (12%), secukinumab (10%), ustekinumab (8%), and infliximab (8%). While approximately 40% of patients persisted on therapy following 20 months of treatment, only about 20% of patients remained on any particular biologic agent after 5 years. Analyzing the data for all treatment periods while taking into account all lines of therapy revealed that secukinumab had a higher persistency than adalimumab, infliximab, and ustekinumab, with a log rank of 0.022, 0.047, and 0.001, respectively. Female sex and smoking were associated with lower drug persistence (HR = 1.25, 95% CI = 1.13–1.38 and HR = 1.109, 95% CI = 1.01–1.21, respectively). On analyzing the data using only the first indicated biologic line, no superiority of any single anti-tumor necrosis factor-alpha (anti-TNFα) agent was observed, while secukinumab was found to be superior as second line therapy to adalimumab, etanercept, infliximab, and ustekinumab but not to golimumab with a log rank *P* value of 0.001, 0.004, 0.025, and 0.002, respectively.

**Conclusions:**

In this large observational cohort studied in the era of biologic therapy, a relatively low drug persistence was observed, with female sex and smoking having a negative impact on persistency. None of the anti-TNFα agents was found to be more persistent than others as first line therapy, while secukinumab was found to be superior to other biologics when indicated as second line of therapy.

## Background

Psoriatic arthritis is a seronegative spondyloarthropathy that greatly impacts patients’ function and quality of life [1]. Beyond the musculoskeletal and skin features of the disease, patients with PsA experience fatigue, physical function limitations, sleep disturbance, and diminished work capacity and social participation [2].

In the past few decades, treatment of PsA has changed based on a better understanding of disease pathogenesis. Targeting TNFα is considered a milestone in the paradigm of PsA treatment as several randomized controlled trials and registries have demonstrated the efficacy of anti-TNFα agents in halting clinical and radiographic progression [3]. More recently, the interleukin-23 (IL-23)/T_h_17 axis has also been shown to play an important role in PsA disease pathogenesis with the IL-12/23 p40 inhibitor (ustekinumab) and IL-17A inhibitors (secukinumab and ixekizumab) also playing a pivotal role in disease control [3]. The most recent 2019 European League Against Rheumatism (EULAR) recommendations for PsA treatment advise on use of either IL-17 inhibitors or TNFα blockers as first line biologic therapy for PsA patients [4].

As more treatment options for PsA are currently available on the market than ever before, describing treatment persistence from real-life experience is gaining in importance as it contributes to better-informed clinical decisions regarding therapeutic choices for PsA patients with the goals of optimizing symptom remission, promoting functional recovery, and reducing healthcare costs.

The aim of this study was to estimate the treatment persistence in a real-world setting of biologic agents prescribed to PsA patients as well as factors associated with improved persistence of these drugs.

## Methods

Clalit Health Services (CHS), Israel’s largest healthcare provider serving some 4.4 million members constituting 52% of Israel’s population, maintains a database that receives continuous real-time input from pharmaceutical, medical, and administrative digital systems [5]. Designed for purposes of administrative and clinical management, the database is available for clinical studies. The quality and accuracy of the register are high, and the reliability of the diagnoses of PsA was estimated in another study and found to be high [6] with a positive predictive value, sensitivity, and specificity of 90.5%, 88.7%, and 88.1%, respectively.

All patients with the diagnosis of PsA above the age of 18 with at least two consecutive dispensed prescriptions with any available biologic agent indicated for PsA under the Israeli National Healthcare Drug Plan from January 1, 2002, until December 31, 2018, were identified and followed until medication stop date or the end of the observation period. Under the Israeli National Healthcare Drug Plan, treatment with biologic therapy for PsA is indicated after the failure of two conventional disease-modifying anti-rheumatic drugs (cDMARDs). At the time of the study, six biologic agents were approved and available on the Israeli market for PsA, including adalimumab, etanercept, infliximab, golimumab, ustekinumab, and secukinumab. Treatment was considered non-persistent whenever a 6-month interval from time of prescription of any biologic agent until the time of medication filling was exceeded provided patients were not started on a different biologic agent during that period, in order to prevent the counting of any short absences of treatment for reasons such as infection or surgery to be classified as non-persistency. This 6-month time interval was decided after analyzing the data to allow for a reasonable grace period for medication filling as a shorter grace period of 3 or 4 months might have resulted in a higher misclassification rate of non-persistence.

Demographic data including age, sex, ethnicity (Jewish or Arab), smoking history (current or past smoking), and socioeconomic status (SES) at inception (determined according to the CHS categories of low, medium, and high, a classification system which has been shown to highly correlate with SES assigned by the Israel Central Bureau of Statistics) [7] were collected. The BMI and patients’ Charlson comorbidity index were calculated from the CHS database. Data regarding the concomitant use of glucocorticosteroids (GC) and cDMARDs such as methotrexate (MTX), leflunomide (LEF), sulfasalazine (SSZ), and hydroxychloroquine (HCQ) were also extracted from the database.

Descriptive statistics, including means (standard deviations) for continuous variables and frequencies (%) for categorical variables, were used. Persistence estimates were derived using non-parametric survival analysis using Kaplan-Meier functions, with treatment discontinuations as failure events. Patients were right-censored for death and end of data availability. Cox regression hazard ratio models were conducted to investigate factors associated with drug persistence including age, sex, smoking status, the Charlson comorbidity index, the use of cDMARDs, and the year of initial availability of each biologic agent on the Israeli market.

The study was approved by the Institutional Review Board of Carmel Medical Center, Haifa (CMC0014-14).

## Results

A total of 2301 PsA patients were identified and included in the analyses after meeting the inclusion criteria of the study. Patients’ characteristics are described in Table 1. The mean age of the study population was 50.9 ± 14 years, 54% were females, 70.4% were with BMI > 25 kg/m^2^, 36% were considered obese (BMI > 30 kg/m^2^), 40% were current smokers, and only 24% had a Charlson comorbidity index of 0.

**Table 1:** Demographics and characteristics of the study group

Patients’ characteristics		***N*** = 2301	
**Age**		50.9 ± 14 years	
**Sex**	Male	429	46.1%
	Female	502	53.9%
**SES**	Unknown	6	0.7%
	Low	275	30.8%
	Medium	365	40.8%
	High	248	27.7%
**Ethnicity**	Arab	120	13.4%
	Jewish	774	86.6%
**BMI**	0–18.5	21	2.3%
	18.5–25	249	27.3%
	25–30	304	33.4%
	30+	337	37.0%
**Obesity**	No	589	63.3%
	Yes	342	36.7%
**Smoking status**	No	557	59.9%
	Yes	373	40.1%
**Charlson comorbidity index**	0	227	24.4%
	1	372	40.0%
	2	148	15.9%
	3	103	11.1%
	4+	80	8.6%

*BMI* body mass index, *SES* socioeconomic status

From 2002 to 2018, a total of 2958 treatment periods were identified. The disposition of the patients and of the treatment periods is described in Table 2. The most commonly prescribed drug was etanercept (33%), followed by adalimumab (29%) and infliximab in only 8% of the cases. Fewer prescriptions of other biologic agents partially due to later entry of these therapeutic agents into the Israeli drug market or due to physician/patient preferences were noted.

**Table 2:** Treatment periods and frequency in the study group

Treatment periods			Patients on biologics		
	Treatment periods	Percentage of patients		Number of patients	Percentage of patients
**Adalimumab**	848	28.7	**Adalimumab**	660	28.7
**Etanercept**	989	33.4	**Etanercept**	643	27.9
**Infliximab**	233	7.9	**Infliximab**	179	7.8
**Ustekinumab**	240	8.1	**Ustekinumab**	199	8.6
**Golimumab**	356	12.0	**Golimumab**	331	14.4
**Secukinumab**	292	9.9	**Secukinumab**	289	12.6
**Total**	2958	100.0	**Total**	2301	100.0

The concomitant use of cDMARDs with bDMARDs is listed in Table 3. A notable decrease in the use of MTX is seen with the introduction of all biologics. However, patients on infliximab were more likely to stay on concomitant treatment with MTX. Oral/parenteral GC were prescribed at least once during 33–43% of the treatment periods.

**Table 3:** The concomitant use of cDMARD/steroid medications with biologics

		Treatment periods												***P*** value
		Adalimumab		Etanercept		Infliximab		Ustekinumab		Golimumab		Secukinumab		
**Concomitant MTX**	**No**	642	75.7%	807	81.6%	159	68.2%	210	87.5%	267	75.0%	244	83.6%	0.001
	**Yes**	206	24.3%	182	18.4%	74	31.8%	30	12.5%	89	25.0%	48	16.4%	
**Concomitant other cDMARDs**	**No**	783	92.3%	919	92.9%	210	90.1%	221	92.1%	311	87.4%	264	90.4%	0.03
	**Yes**	65	7.7%	70	7.1%	23	9.9%	19	7.9%	45	12.6%	28	9.6%	
**MTX before**	**No**	163	19.2%	177	17.9%	47	20.2%	43	17.9%	44	12.4%	40	13.7%	0.03
	**Yes**	685	80.8%	812	82.1%	186	79.8%	197	82.1%	312	87.6%	252	86.3%	
**cDMARDs before**	**No**	322	38.0%	319	32.3%	106	45.5%	103	42.9%	101	28.4%	102	34.9%	0.001
	**Yes**	526	62.0%	670	67.7%	127	54.5%	137	57.1%	255	71.6%	190	65.1%	
**GC**	**No**	510	60.1%	633	64.0%	132	56.7%	141	58.8%	195	54.8%	196	67.1%	0.03
	**Yes**	338	39.9%	356	36.0%	101	43.3%	99	41.3%	161	45.2%	96	32.9%	

*cDMARDs* conventional disease-modifying anti-rheumatic drugs, *GC* glucocorticosteroids, *MTX* methotrexate

**Fig. 1:**
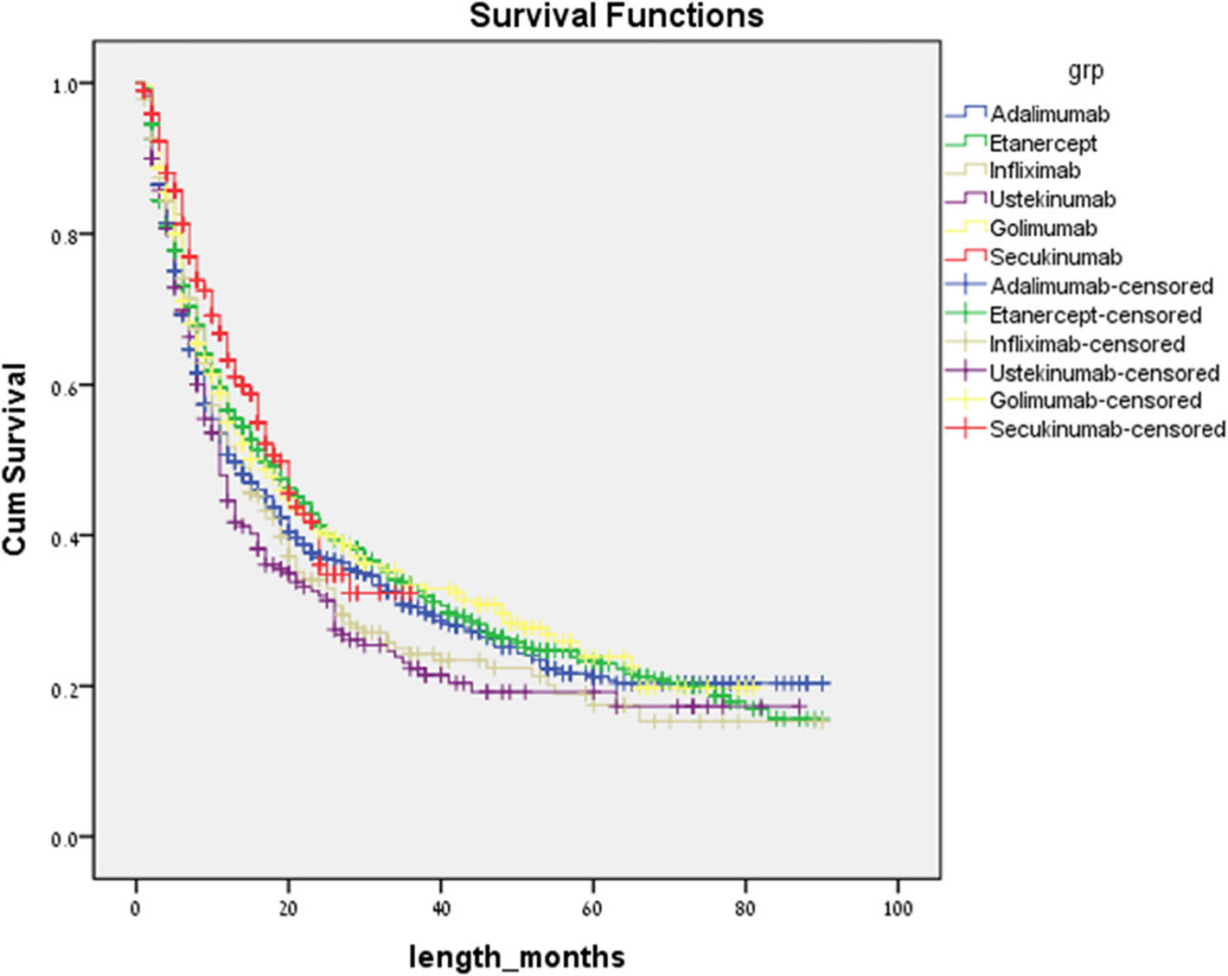
Unadjusted time to non-persistence for all biologics used as all lines of therapy

A Kaplan-Mayer survival analysis with pairwise comparisons of all treatment choices with respect to lines of therapy was conducted. When analyzing the data for all treatment periods while taking into account all lines of therapy, secukinumab had a higher persistency than adalimumab, infliximab, and ustekinumab, with a log rank of 0.022, 0.047, and 0.001, respectively, as is shown in Fig. 1. Comparisons of other treatment modalities were not significantly different in this model.

**Fig. 2:**
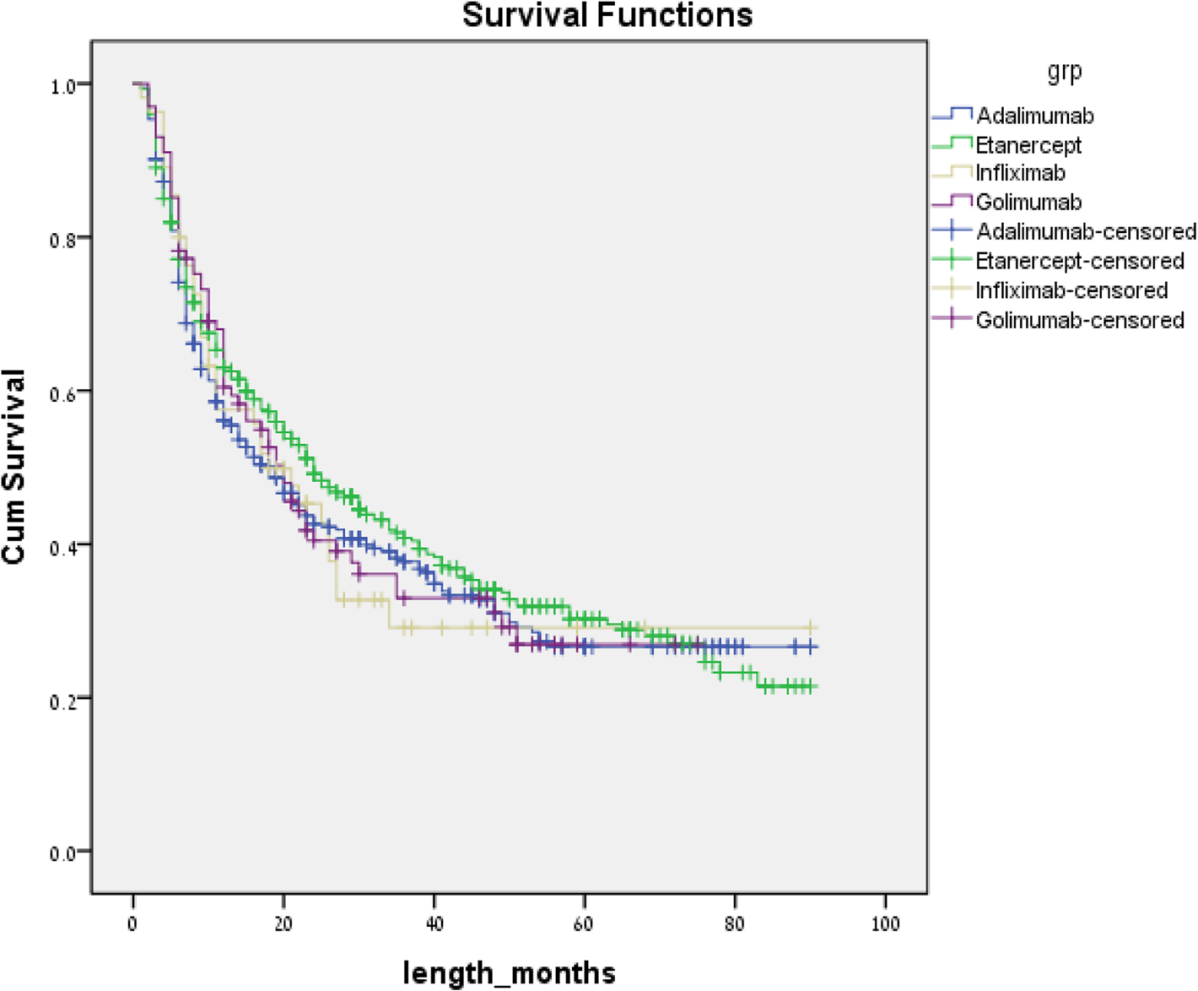
Unadjusted time to non-persistence for biologics used as first lines of therapy

On analyzing the data using only the first indicated biologic line, no biologic agent was found to be superior to any other as is shown in Fig. 2. Of note, during the study period, the four TNF-alpha blockers etanercept, adalimumab, infliximab, and golimumab were the only medications of this class available to be used as first line agents on the Israeli market so the comparison did not include secukinumab or ustekinumab.

**Fig. 3:**
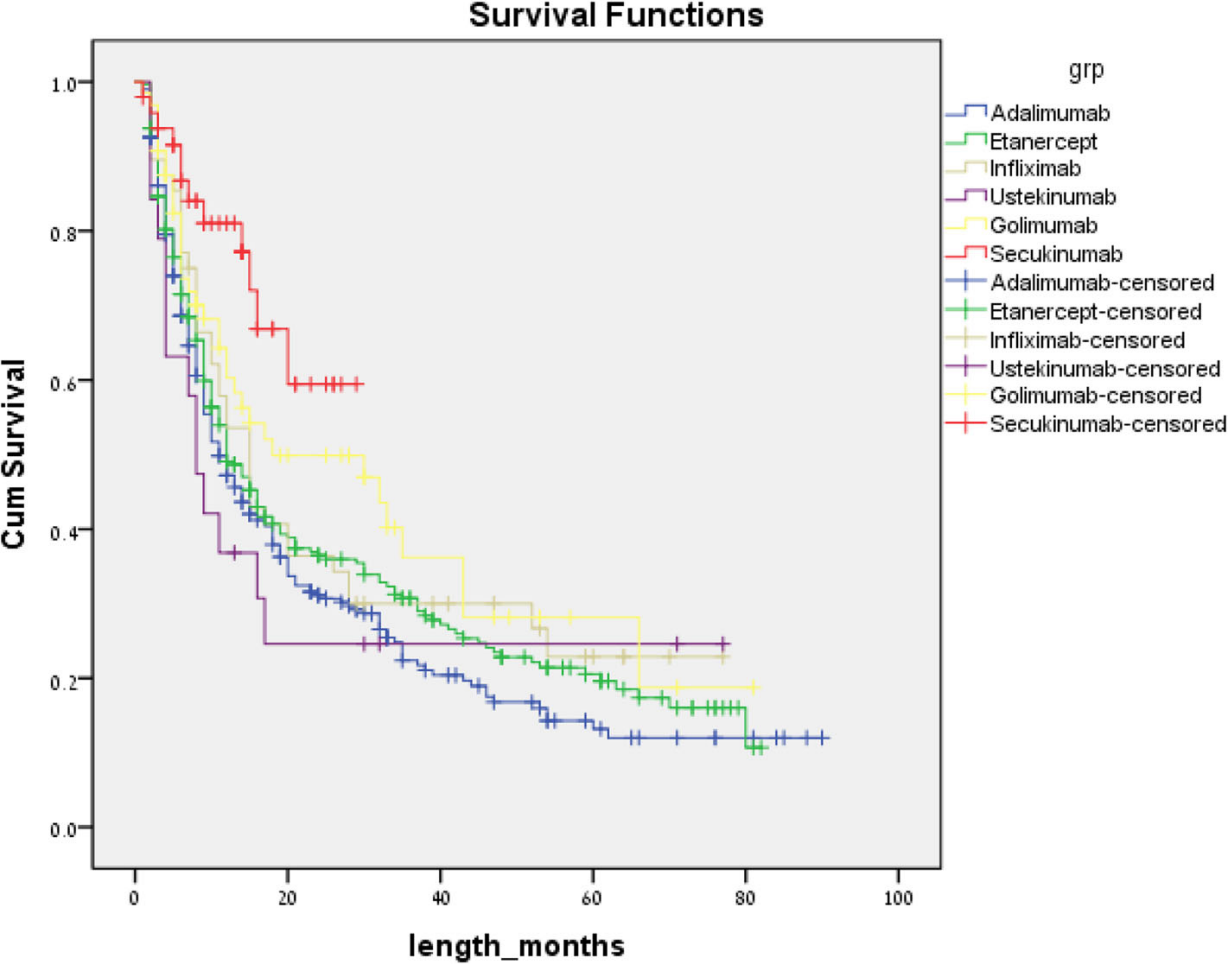
Unadjusted time to non-persistence for biologics used as second line of therapy

As all biologic agents were equally available for use as second line agents, a survival analysis was conducted censoring for medications used as second line agents, with results shown in Fig. 3. The analysis revealed superiority of secukinumab compared to adalimumab, etanercept, infliximab, and ustekinumab but not to golimumab with a log rank *P* value of 0.001, 0.004, 0.025, and 0.002, respectively. Golimumab had a higher persistency than adalimumab with a log rank *P* value of 0.027. Other pairwise comparisons did not reach statistical difference.

Cox regression hazard ratio models were conducted to investigate factors associated with drug persistence as shown in Table 4. Female sex and smoking were associated with lower drug persistence. Patients who were obese also had lower estimates for drug persistency although not reaching statistical significance. Patients who were on concomitant treatment with MTX or GC were more likely to stay persistent on their biologic therapy. Moreover, a negative correlation was noted between drug persistency and the advancement of the various treatment lines, with a gradient decrease in drug persistency as patients advanced through alternative lines of biologic therapy, so that patients failing prior lines of treatment were less likely to stay persistent on newer lines of therapy.

**Table 4:** Adjusted Cox proportional hazard model for treatment discontinuation

	HR	95.0% CI	
		Lower	Upper
Female vs. male	1.249	1.134	1.376
Age 30–39 (compared to 18–29)	0.972	0.785	1.204
Age 40–49	1.000	0.810	1.234
Age 50–59	1.032	0.836	1.273
Age 60–69	0.945	0.758	1.178
Age > 70	1.061	0.812	1.387
SES—medium	0.898	0.798	1.011
SES—high	0.918	0.805	1.048
Ethnicity—Arab	1.136	0.972	1.328
Ethnicity—Jewish	0.913	0.693	1.203
Charlson comorbidity index	1.004	0.973	1.035
BMI_thin	1.146	0.833	1.577
BMI_overweight	1.082	0.952	1.228
BMI_obese	1.126	0.993	1.278
Smokers	1.109	1.008	1.219
Concomitant MTX	0.830	0.737	0.933
Concomitant cDMARDs	0.958	0.811	1.131
MTX use before	0.891	0.788	1.008
cDMARDs use before	1.027	0.931	1.134
Concurrent GC	0.569	0.515	0.629
Rx line2 vs. line1	1.263	1.117	1.427
Rx line3 vs. line1	1.296	1.119	1.500
Rx line4 vs. line1	1.590	1.382	1.829
Start of biologic 2012 vs. 2018	1.657	1.339	2.051
Start of biologic 2013 vs. 2018	1.526	1.234	1.887
Start of biologic 2014 vs. 2018	1.484	1.204	1.829
Start of biologic 2015 vs. 2018	1.521	1.233	1.878
Start of biologic 2016 vs. 2018	1.697	1.393	2.066
Start of biologic 2017 vs.2018	1.410	1.157	1.719

*BMI* body mass index, *cDMARDs* conventional disease-modifying anti-rheumatic drugs, *GC* glucocorticosteroids, *MTX* methotrexate, *Rx* treatment, *SES* socioeconomic status

Because time of introduction of any particular biologic agent into the Israeli drug market could potentially affect treatment choices and decisions, we incorporated the year of initial medication arrival onto the Israeli market into the statistical model, and compared it to 2018. In this analysis, we did not find any preference for any one medication over other medications for any given year. The 1-year, 2-year, and 5-year survival rate for biologics used in all lines of therapy ranging between 44.6–63.2%, 31.9–41.7%, and 17.3–23.5% as reported in Table 5.

**Table 5:** The overall survival of biologic DMARDs

	1-year survival (%)	2-year survival (%)	5-year survival (%)
**Adalimumab**	**50.7**	**37**	**21.3**
**Etanercept**	**56.4**	**41.2**	**23.3**
**Infliximab**	**51.7**	**34**	**17.5**
**Golimumab**	**55**	**40.5**	**22.5**
**Ustekinumab**	**44.6**	**31.9**	**17.3**
**Secukinumab**	**63.2**	**41.7**	**–**

## Discussion

Real-world data on drug persistency in PsA patients is scarce. This study was conducted from a large, comprehensive, national dataset, composed of a diverse general population of 2301 PsA patients using all available bDMARDs indicated for PsA in Israel from 2002 until 2018.

We report higher drug persistency rates in PsA patients on secukinumab when indicated as second line therapy compared to adalimumab, infliximab, and ustekinumab. Our study also demonstrates improved biologic drug persistency in male PsA patients over female patients, with a reduction in drug persistency associated with smoking as well as biologic monotherapy.

Our real-world results on biologic persistence are in keeping with a study by Stober et al. [8] which evaluated the persistency of TNFα blockers limited to etanercept and adalimumab in 188 PsA patients, showing a lower persistency in female patients. As in that study, we did not find improved drug survival when analyzing the data for the first biologic agent used, which included the four TNFα blockers available on the market at the time of the study. As for the effect of MTX in combination therapy, we report a better drug persistency in patients on concurrent MTX. The role of MTX has been controversial, with a systemic review on all TNFα blockers failing to show any effect of combination therapy on drug survival except for infliximab [9], and with the SEAM study reporting no advantage for combination therapy of MTX with etanercept over monotherapy with etanercept alone [10]. However, drugs targeting the IL-17 pathway had similar efficacy in monotherapy or in combination with MTX as was shown in the FUTURE2, SPIRIT H2H, and EXCEED clinical trials data [11–13].

Our study did not find any association between patient age and SES on drug persistency. Our finding that smoking is a predictor for non-persistency is supported by other observational studies in the literature [14–16], as tobacco use is considered a significant environmental risk factor for developing inflammatory arthritis and there are indications that smoking exacerbates the symptoms and worsens disease outcomes. As for obesity, evidence from the literature points to its being a major culprit for disease non-response [17]. Findings from our study also support this notion, as there was a trend for lower drug persistency in obese patients, but this finding did not reach statistical significance.

As expected, patients who failed prior biologic therapy were less likely to remain persistent on newer biologic agents, in keeping with the report of Harrold et al. on data from the Corrona Registry on 549 biologic-naïve and 692 biologic-experienced PsA patients showing a greater mean time to non-persistence in the former group [18]. In addition, numerous studies have demonstrated that switching between TNFα blockers results in lower response rates than when used as first line therapy [19, 20].

Our study showed that the persistence of golimumab was better than adalimumab when censoring the data for the second biologic line used. This is in keeping with the results of a study by Rotar et al. [21] on 2022 patients with either rheumatoid arthritis (RA), ankylosing spondylitis (AS), or PsA, which showed better persistence of golimumab compared to other TNFα blockers in biologic-experienced AS and PsA patients but not overall.

Our results report a 1-year, 2-year, and 5-year survival rate for biologics used in all lines of therapy ranging between 44.6–63.2%, 31.9–41.7%, and 17.3–23.5%, respectively, marginally lower compared to previous reports. In our review of the literature, the definitions used for drug persistency vary considerably in different studies. Saad et al. [22] showed that approximately 82%, 70%, and 59% of the population from the British Society of Rheumatology Biologics Register involving 566 participants with PsA remained on the first anti-TNFα agent after 1, 2, and 3 years of treatment, respectively. In 2016, Palmer and colleagues [23] observed that the mean biologic drug persistence was approximately 17 months among 990 PsA patients receiving TNFα blockers as first line therapy. In a different study, Fagerli et al. [24] estimated that approximately 47% of 625 participants diagnosed with PsA from the UK remained on their initial anti-TNFα therapy after 5 years. Another study from an administrative claims database in the USA [25] on 1235 PsA patients reported 56% of patients discontinuing their index biologic agent within a year, with a mean duration of persistence of 8 months. More recently, Jacob and colleagues [26] reported a low persistence of biologics of 32% after 5 years of follow-up on PsA patients in rheumatology practices in Germany. Yet another recent study by the EuroSpa collaboration registries from Europe reported a 1-year retention rate of 77% for TNFα inhibitors in over 14,000 biologic-naïve PsA patients compared to a 2-year drug survival of 56% and 50% for PsA patients on second line biologic therapy in the NOR-DMARD study [27] and in the Portuguese registry [28], respectively. Of note, it is important to emphasize that the definition of persistency in these studies was not similar, and the study populations, insurance coverages, and line of treatment reported differ as well precluding a direct comparison among the studies.

In our study, secukinumab had a better persistency when indicated as second line therapy. A number of matching-adjusted indirect comparisons have been published demonstrating conflicting though higher responses for secukinumab over infliximab [29], adalimumab [30], and etanercept [31]. Notably, secukinumab only narrowly missed statistical significance for superiority over adalimumab in the primary endpoint of the head-to-head EXCEED trial, showing only numerically higher results versus adalimumab [13]. Thus, our data might suggest that switching to secukinumab would be a better medication choice after TNFα blocker failure. This is more in line with the 2015 Group for Research and Assessment of Psoriasis and Psoriatic Arthritis (GRAPPA) treatment recommendations [3] and the updated 2019 EULAR recommendations [4] which place secukinumab as a first line agent alongside other biologics, unlike the earlier 2015 EULAR [32] as well as the 2018 American College of Rheumatology (ACR)/National Psoriasis Foundation (NPF) [33] guidelines, which specified TNFα blockers as first line biologic therapy and anti-IL-17 agents to be considered when TNFα blockers are “inappropriate.”

There are some limitations in our study. Our results should be interpreted in caution as treatment indications and switching were not based on any protocol but rather on patient-physician choices and preferences. Moreover, we did not have any information on disease activity parameters involving joints and skin, nor any data on side effects that could have warranted medication switch and might have shed more light on the reason for treatment change and relatively low persistency rates.

On the other hand, the strengths of our study lie in our large dataset on PsA patients, providing a real-life image of drug treatment patterns and persistency as well as predictors for drug persistency.

In summary, our study of a large observational PsA cohort found a relatively low persistence of biologic therapy in rheumatology practices, with female sex and tobacco use having a negative impact on drug persistency, and secukinumab being superior to other biologic agents when indicated at second line of therapy. These findings can improve treatment planning and provide a more efficient allocation of societal economic resources. However, further studies are needed to establish the role and level of anti-IL-17 agents in the PsA treatment paradigm and to improve our understanding of the reasons for non-persistence.

## Data Availability

All data generated or analyzed during this study are included in this published article.

## Abbreviations

CASPAR: Classification Criteria for Psoriatic Arthritis
cDMARDs/bDMARDs: Conventional/biologic disease-modifying anti-rheumatic drugs
IL: Interleukin
PsA: Psoriatic arthritis
TNFα: Tumor necrosis factor-alpha
